# Medical and Psychosocial Challenges Associated with Breast Cancer Survivorship

**DOI:** 10.7759/cureus.13211

**Published:** 2021-02-07

**Authors:** Raja Rahool, Ghulam Haider, Aisha Shahid, Mehwish R Shaikh, Paras Memon, Bhunisha Pawan, Shumyla Beg, Kiran Abbas, Manahil Khalid

**Affiliations:** 1 Oncology, Jinnah Postgraduate Medical Centre, Karachi, PAK; 2 Internal Medicine, Jinnah Postgraduate Medical Centre, Karachi, PAK; 3 Internal Medicine, Bahria University Medical and Dental College, Karachi, PAK

**Keywords:** breast neoplasms, financial stress, survivorship, social support, mastectomy, psycho-oncology, psychophysiology, cancer survivors

## Abstract

Objective

To assess the association between common survivorship issues and characteristics of breast cancer survivors presenting at a tertiary care hospital in Karachi, Pakistan.

Methodology

This study was conducted in the medical oncology department of Jinnah Medical Postgraduate Center from March 27, 2019, to September 27, 2019. A number of 257 females of age group 18-90 years who had either completed their treatment or were undergoing treatment at the time were included using non-probability consecutive sampling techniques. Face-to-face interviews were personally conducted by the researcher, and data regarding the socio-demographics and common survivorship issues faced by breast cancer patients were obtained. The data acquired were entered and analyzed using SPSS version 23 (IBM Corp, Armonk, NY).

Results

The mean age of the breast cancer survivors were 42.58 ± 10.07 years. Of the main challenges, lack of energy received the highest mean score of 3.44 ± 1.26, followed by fatigue and financial issues. Overall the most common survivorship issue were financial issues (81.3%), followed by fatigue (80.9%), cessation of the menstrual cycle (66.1%), weak social support (59.1%), and cosmetic disfigurement (51.8%).

Conclusion

Breast cancer survivors have various psychological, medical, and social issues and may require unique attention during follow-up visits.

## Introduction

One of the most prevalent malignancies among females is breast cancer. Globally, in 2018 the incidence of breast cancer was estimated as 25% of all cancers [1]. Within the last 10 years, the incidence of breast cancer has increased by 6%, mortality rates have decreased significantly and at present, 80% of early breast cancer patients survive for more than 10 years [2]. In 2010, there were an estimated 500,000 breast cancer survivors and by 2040 it is expected to cross two million survivors [3]. The five-year survival rate of breast cancer differs from country to country. In India, the survival rate was estimated as 60%, in Malaysia as 68%, in South Africa as 53%, in Mongolia as 57% and in Oceania and North America up to 89% [4]. 

The rate of survival of breast cancer patients has been significantly enhanced with improvements and availability of therapy. However, literature has shown that women with breast cancer who have undergone chemotherapy, radiotherapy, biotherapy or hormonal therapy, surgery, and reconstructive surgery may face numerous psychosocial and physical issues and as well as a compromised overall quality of life [5]. 

After adjuvant therapy, most of the women encounter weight gain, musculoskeletal pain, hot flashes, nausea, and itching around the genital area. [6] The sexual desire of breast cancer survivors is also highly affected after therapy and may experience fatigue, loss of nipple sensitivity, vaginal dryness, and scarring [6]. Psychological problems such as sadness, anxiety, and depression owing to therapy, recurrence, and bodily disfigurement stay constant after diagnosis and cure. Besides, the lack of support and communication from their partners, family, and friends reduced their social integration [5, 7]. Survivors of breast cancer are also at high risk of secondary health issues such as cardiovascular disease, cessation of the menstrual cycle, and poor bone health (osteoporosis) [8]. Other common issues are low physical activity and poor dietary habits, which are associated with recurrence of breast cancer [9]. The majority of breast cancer survivors are unable to rejoin their respective occupations due to their inability to work. This is associated with a high financial burden because the resources to pay consultation fees, medical bills, and medications become limited, therefore they show poorer medication adherence [10].

As the survival rate of breast cancer is increasing, the long-term social, physical, and psychological issues are also becoming very common among them. There is no data available in Pakistan addressing the most common issues which have a significant impact on the quality of their life. So, the current research aimed to assess the common survivorship issues faced by breast cancer patients. This research would be helpful for health professionals to better understand the needs of recovered patients, identify issues and develop strategies to resolve them so that women can have a good quality of life. 

## Materials and methods

It was a cross-sectional study performed at the department of the medical oncology department of Jinnah Medical Postgraduate Center from March 2019 to September 2019. The sample size of 257 was estimated using the OpenEpi online sample size calculator by taking proportion as 0.28 [5] of vaginal dryness among breast cancer survivors, absolute precision as 5.5%, and 95% confidence level. All the females of age 18-90 years who had completed their treatment and who were on treatment were included using the non-probability consecutive sampling technique. Women with psychiatric problems, having pregnancy, a history of recurrent disease, and who didn’t give consent were excluded from the study.

Approval from the hospital's ethical review committee was sought before data collection and informed written or verbal consent was taken from all the eligible females. Face-to-face interviews were personally conducted by the researcher and information was noted in the questionnaire while maintaining the confidentiality and privacy of data. 

The questionnaire was divided into two parts, the first part included the questions regarding the socio-demographic properties of the participants and the second part included the questions regarding the common survivorship issues faced by breast cancer patients. The issues were evaluated using "Breast cancer prevention trial symptoms scale (BCPT)" and "Cancer rehabilitation evaluation system (CARES)" [11-13]. The BCPT scale assessed the symptoms such as fatigue, nausea, hot flashes, cognitive problems, musculoskeletal pain, weight problems, vaginal problem, bladder problem, arm problems, and sexual interest whereas the CARE scale was used to evaluate the psychological issues. Furthermore, medical issues such as any cardiovascular problem, hormonal blockade, and osteoporosis within the last one month were assessed using the medical history of the patients. The questions regarding the weak family support, financial strain and cosmetic issues were also asked. The females reported the severity of each problem experienced by them in the last one month on a five-point Likert scale of 0 (not all) to 4 (extremely). The cutoff scores for bladder and vaginal problems, as well as other medical and social issues, were taken as ≥4; for hot flashes, weight and arm problems, fatigue and sexual interest as ≥6; for musculoskeletal and cognitive problems as ≥8, and for psychological problems as ≥12. The final questionnaire was translated into Urdu and pre-testing of the final questionnaire was done in 10 survivors of breast cancer and Cronbach’s alpha value was calculated as 0.72. 

All the data was entered and analyzed using SPSS version 23 (IBM Corp., Armonk, NY). Quantitative variables were described as mean and SD, while qualitative variables were displayed as frequency and proportion. The common survivorship issues were stratified with respect to age, residence, socioeconomic status, education, and marital status. The post-stratification chi-square test was applied. The p-value<0.05 was considered as statistically significant. 

## Results

The mean age of the 257 breast cancer survivors was 42.90 ± 10.39 years. The mean BMI was reported as 26.90±5.96 kg/m2. The majority of the patients were living in urban areas (73.9%) whereas 67 were from rural areas (26.1%). Out of 257, most of them were illiterate (n=90; 35%) followed by primary (n=81; 31.5%), secondary (n=49; 19.1%), intermediate (n=22, 8.6%) and graduate (n=15, 5.8%). Also, 185 (72%) had low socioeconomic status (monthly income <15,000 rupees), 67 (26.1%) had middle socioeconomic status (monthly income 15,000-30,000 rupees) and only five (1.9%) had high socioeconomic status (monthly income >30,000 rupees). The majority of the females were married (n=210, 81.7%) whereas 47 (18.3%) were unmarried. Individual responses and mean scores of breast cancer survivorship issues by study participants are displayed in Table 1. 

**Table 1:** Mean scores and distribution of breast cancer survivorship issues in study participants.

Survivorship Issues	Not at all (0)	A little (1)	Fair (2)	Much (3)	Very much (4)	Mean ± SD
Hot flashes						
a. Hot flashes	74(28.8%)	7(2.7%)	37(14.4%)	38(14.8%)	101(39.3%)	2.33 ± 1.67
b. Night sweats	116(45.1%)	4(1.6%)	18(7.0%)	37(14.4%)	82(31.9%)	1.86 ± 1.8
Bladder control						
Loss of bladder control	175(68.1%)	4(1.6%)	11(4.3%)	14(5.4%)	53(20.6%)	1.09 ± 1.67
Vaginal problems						
Vaginal dryness	108(42%)	9(3.5%)	52(20.2%)	16(6.2%)	72(28%)	1.75 ± 1.69
Musculoskeletal problems						
a. General aches and pain	71(27.6%)	2(0.8%)	8(3.1%)	15(5.8%)	161(62.6%)	2.75 ± 1.77
b. Joint pain	188(73.2%)	5(1.9%)	0(0.0%)	4(1.6%)	60(23.3%)	1 ± 1.7
c. Muscle stiffness	194(75.5%)	5(1.9%)	0(0.0%)	4(1.6%)	54(21%)	0.91 ± 1.65
Weight problems						
a. Unhappy with appearance of owns body	139(54.1%)	10(3.9%)	22(8.6%)	20(7.8%)	66(25.7%)	1.47 ± 1.74
b. Weight gain	169(65.8%)	2(0.8%)	6(2.3%)	15(5.8%)	65(25.3%)	1.24 ± 1.77
Cognitive problems						
a. Forgetfulness	166(64.6%)	8(3.1%)	7(2.7%)	18(7%)	58(22.6%)	1.2 ± 1.72
b. Difficulty concentrating	153(59.5%)	21(8.2%)	10(3.9%)	6(2.3%)	67(26.1%)	1.27 ± 1.74
c. Easily distracted	185(72%)	5(1.9%)	11(4.3%)	15(5.8%)	41(16%)	0.92 ± 1.56
Arm problems						
a. Decreased range of motion of affected arm	186(72.4%)	6(2.3%)	7(2.7%)	11(4.3%)	47(18.3%)	0.94 ± 1.6
b. Arm swelling	203(79%)	6(2.3%)	10(3.9%)	9(3.5%)	29(11.3%)	0.66 ± 1.37
Fatigue						
a. Tiredness	26(10.1%)	0(0.0%)	9(3.5%)	43(16.7%)	179(69.6%)	3.36 ± 1.23
b. Lack of energy	33(12.8%)	0(0.0%)	3(1.2%)	24(9.3%)	197(76.7%)	3.37 ± 1.34
Sexual interest						
a. Lack of interest in sex	73(28.4%)	28(10.9%)	33(12.8)	18(7%)	105(40.9%)	2.21 ± 1.71
b. Low sexual enjoyment	96(37.4%)	28(10.9%)	36(14%)	44(17.1%)	53(20.6%)	1.73 ± 1.59
Psychological problems						
a. Felt worried and anxious about future	85(33.1%)	5(1.9%)	8(3.1%)	31(12.1%)	128(49.8%)	2.44 ± 1.81
b. Felt despair about health	71(27.6%)	0(0.0%)	10(3.9%)	27(10.5%)	149(58%)	2.71 ± 1.74
c. Felt sad depressed and lost interest	119(46.3%)	17(6.6%)	29(11.3%)	31(12.1%)	61(23.7%)	1.6 ± 1.69
d. Felt angry and irritable	123(47.9%)	10(3.9%)	27(10.5%)	29(11.3%)	68(26.5%)	1.65 ± 1.74
Other medical issues						
a. Cardiovascular issue	184(71.6%)	31(12.1%)	18(7%)	4(1.6%)	20(7.8%)	0.62 ± 1.19
b. Cessation of menstrual cycle	56(21.8%)	18(7.0%)	8(3.1%)	5(1.9%)	170(66.1%)	2.84 ± 1.71
c. Osteoporosis	136(52.9%)	35(13.6%)	14(2%)	2(0.8%)	70(27.2%)	1.36 ± 1.72
Other social issues						
a. Financial issue	36(14%)	7(2.7%)	3(1.2%)	2(0.8%)	209(81.3%)	3.33 ± 1.45
b. Weak family support	68(26.5%)	5(1.9%)	12(4.7%)	24(9.3%)	148(57.6%)	2.7 ± 1.73
c. Cosmetic issue	110(42.8%)	3(1.2%)	6(2.3%)	5(1.9%)	133(51.8%)	2.19 ± 1.95

**Figure 1:**
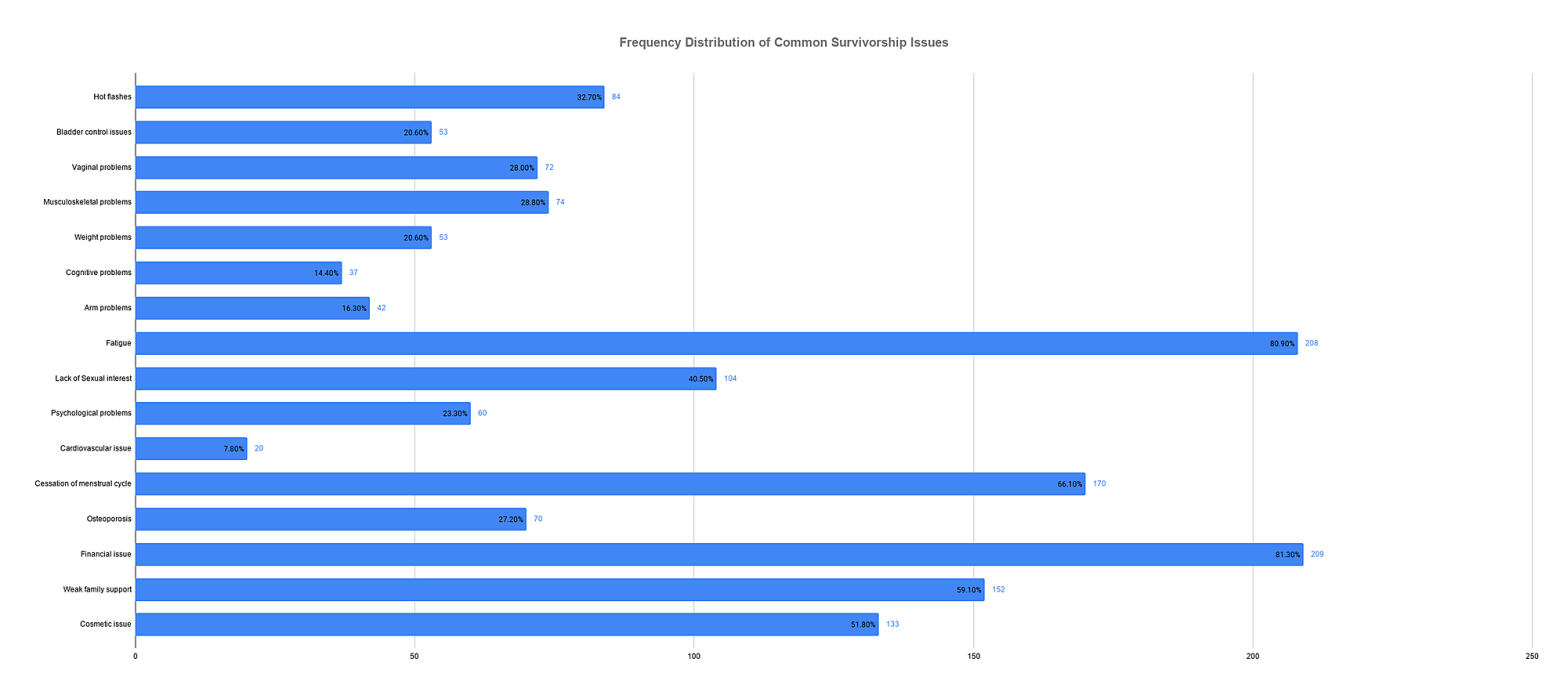
Frequency of Common Survivorship Issues

The lack of energy recorded the highest mean score at 3.44 ± 1.26, followed by fatigue and financial issues. Overall the most common survivorship issue was financial issues (n=209; 81.3%), followed by fatigue (n=08; 80.9%), cessation of menstrual cycle (n=170; 66.1%), weak social support (n=152; 59.1%), and cosmetic disfigurement (n=133; 51.8%) as displayed in Figure 1.

Stratification with respect to age, residence, educational status, socioeconomic status, and marital status was done to see the effect on survivorship issues. The issue of hot flashes showed a statistically significant relationship with age, educational status, and marital status (p<0.05). Bladder problems showed a statistically significant relationship with age, residence, and marital status (p<0.05). The vaginal, musculoskeletal, and weight problems, as well as cessation of menstrual cycle showed a statistically significant relationship with age, residence, and educational status (p<0.05). Cognitive and arm problems showed a statistically significant relationship with age and residence (p<0.05). Fatigue showed a statistically significant relationship with age, educational status, socio-economic status, and marital status (p<0.05). Lack of sexual interest was statistically significant with respect to age and marital status (p<0.05). Psychological issues showed a statistically significant relationship with educational status (p<0.05). Osteoporosis showed a statistically significant relationship with residence and educational status (p<0.05). Financial stress showed a statistically significant relationship with educational status, socio-economic status, and marital status (p<0.05). Weak social support showed a statistically significant relationship with educational status and socio-economic status (p<0.05). Cosmetic disfigurement showed a statistically significant relationship with educational status (p<0.05). More stratifications are mentioned in Table 2.

**Table 2:** Stratification of Common Survivorship Issues with Patient Characteristics HF=Hot flash, BP=Bladder problem, VP=Vaginal problem, MP=Musculoskeletal problem, WP=Weight problem, CP=Cognitive problem, AP= Arm problem, F=Fatigue, SI=Sexual interest, PI=Psychological issues, CI=Cardiac issues, O=Osteoporosis, CM=Cessation of menstrual cycle, FS=Financial stress, WS=Weak social support, CD=Cosmetic disfigurement,  * = significant p<0.05

	HF	BP	VP	MP	WP	CP	AP	F	SI	PI	CI	O	CM	FS	WS	CD
Age Groups																
<45 years	53	21	58	38	43	19	19	122	82	37	12	41	127	127	89	86
≥45 years	31	32	14	36	10	18	23	86	22	23	8	29	43	82	63	47
P-value	0.778	0.001*	0.001*	0.027*	0.001*	0.155	0.015*	0.029*	0.001*	0.971	0.858	0.506	0.001*	0.448	0.188	0.34
Residence																
Rural	62	46	47	61	32	36	25	153	76	39	12	58	115	152	108	99
Urban	22	7	25	13	21	1	17	55	28	21	8	12	55	57	44	34
P-value	0.975	0.017*	0.049*	0.048*	0.012*	0.001*	0.02*	0.779	0.797	0.072	0.139	0.046*	0.001*	0.359	0.206	0.848
Educational status																
Illiterate	20	15	27	15	27	16	12	67	31	21	8	15	55	72	56	50
Primary	10	5	11	6	3	0	0	7	9	1	2	6	15	8	2	13
Secondary	11	13	15	13	10	11	12	43	26	18	6	15	27	39	31	18
Intermediate	33	18	16	35	13	8	12	74	30	15	4	32	60	74	53	38
Graduate or above	10	2	3	5	0	2	6	17	8	5	0	2	13	16	10	14
P-value	0.005*	0.336	0.001*	0.039*	0.003*	0.046*	0.093	0.010*	0.098	0.044*	0.218	0.022*	0.027*	0.017*	0.011*	0.001*
Socioeconomic status																
<15,000 rupees	61	34	53	58	44	28	29	153	69	50	13	49	121	168	137	99
15,000-30,000 rupees	19	18	19	14	8	9	12	53	33	9	7	19	44	41	15	33
>30,000 rupees	4	1	0	2	1	0	1	2	2	1	0	2	5	0	0	1
P-value	0.06	0.339	0.37	0.23	0.121	0.615	0.892	0.051*	0.232	0.078	0.54	0.776	0.271	0.001*	0.001*	0.299
Marital Status																
Unmarried (n=22)	27	18	9	10	8	6	11	43	1	8	4	9	28	43	33	22
Married (n=98)	57	35	63	64	45	31	31	165	103	52	16	61	142	166	119	111
P-value	0.001*	0.001*	0.134	0.208	0.5	0.725	0.147	0.042*	0.001*	0.257	0.837	0.168	0.292	0.048*	0.088	0.453

## Discussion

Most survivors after and during breast cancer treatment delight in their longer lifespan, but there is currently little knowledge accessible from their view about the most prevalent and important problems they face. Hence, the current study explored the challenges faced by breast cancer survivors and its association with different factors. 

In Asia, developing countries have overcrowded and scarce resources in public hospitals so patients mostly prefer private hospitals for better management. Therefore, the treatment and follow-up is very costly. The majority of the breast cancer survivors struggle with financial stress [14]. In a study by Pietrangelo, about 36% of the women reported being unable to continue their job and lose their careers after surviving breast cancer [15]. In the current study financial issues were the most frequent challenge associated with survivorship among breast cancer patients. The financial constraints were associated with the educational status, socioeconomic background, and marital status of the survivors (p<0.05). In the results of the longitudinal research on breast cancer survivors, it was shown that most of them encountered economic hardships, where out of pocket expenses were 40% among participants with < $500 expenditure [16]. In another research by Fenn in the US, 18% of breast cancer survivors had financial issues and showed a statistically significant association with family income (p<0.05) [17]. A study conducted in Pakistan showed that 35% of the breast cancer survivors belonged to low socioeconomic groups and had five-year disease-free and overall survival of 31% and 45% respectively (p<0.05) [18].

Another challenge faced by survivors was chronic fatigue ranging from 4% to 91%, mainly influencing the quality of life. Fatigue was noted in 50% of the patients after the first five years of therapy [19]. This was in accordance with our study. In a meta-analysis, the prevalence of severe fatigue was reported as 7-52% in breast cancer survivors with pooled prevalence as 27% [20].

The hormonal therapies have shown a significant reduction in the rate of mortality and recurrence rate by 30% and 31% respectively [21]. However, the most common side effects of these therapies are reported as vaginal dryness and hot flashes. In addition, it is also associated with the cessation of the menstrual cycle [22]. In the current research, vaginal dryness, and cessation of menstrual cycle were reported in association with age, residence, and education level (p<0.05). In previous research, almost half of the breast cancer survivors had reported at least one hot flash in the past 1 day (45%) or past seven days (52%) and the majority of them had mild to asymptomatic severity [23]. In another similar research by Sapkota et al. conducted in Nepal, the frequency of hot flashes was reported as 45% and the mean rank for hot flashes was higher in females who had completed their treatment within 1 year. In addition, the percentage of vaginal problems was reported as 28% among them [5].

In Asia, heavily patriarchal culture is present, the final verdict is made by the family's head. Their assistance and choice to go for therapy is therefore very important in breast cancer management. In such situations, if the family is not supportive, patients suffer from psychosocial distress and mental complications [7]. Therefore, strong emotional & social support from family and friends play a vital role in the life of breast cancer survivors. In Pakistan, a study by Banning et al. showed that initially, family support is present however later on it becomes inconsiderate for the patient's family. The patients showed more concerns regarding their children, husband, and family, rather than their own selves [24]. In the current research, weak social support was observed in 59.1% of the females and it was more in low socioeconomic status and illiterate females (p<0.05). In the previous study, social problems were one of the significant issues among breast cancer survivors and had a high mean rank score of 23.9 among women of age <45 years [5]. This is similar to the present study, young women (<45 years) had a high frequency of weak social support. 

Breast cancer treatment involves mastectomy that has side effects such as scarring, weight gain, body dysfunction, and cosmetic disfigurement that have universal female sexuality and womanhood connotations. The change in body image often leads to dissatisfaction and bodily shame and low self-esteem [9]. Bodily disfigurement and sexual dysfunction have a major influence on psychological as physiological aspects of breast cancer survivors. It also affected the sex life of their partners [9]. As young breast cancer patients are more sexually active, lack of sexuality plays an important role [25]. The intrapsychic capacity of females changes due to a sexual dysfunction that involves negative body image, fear of fertility loss, loss of femininity, absence of sexual interest, and shifts to a sense of sexual self [25]. In the present study, the majority of the breast cancer survivors lack their interest in sex due to sexual dysfunction and it was prevalent among age groups less than 45 years (p<0.05). The married females had more sexual dysfunctionality as compared to unmarried and showed statistically significant differences (p<0.05). Furthermore, the frequency of cosmetic disfigurement was 51.8%. In a previous study, the breast cancer survivors reported poor sexual dysfunction and sexual satisfaction as 45% and 44% respectively. About 81% of them showed less sexual sense/desire and 43% of them presented with dyspareunia. Furthermore, 42% of them had anxiety and 44% had depression which was significantly associated with body image (p<0.05) [25]. 

## Conclusions

The present study offers insight into the challenges faced by survivors of breast cancer. It is important to keep a strict follow-up after completion of treatment. The clinicians should assess whether the survivors need counseling from a professional psychologist or any lifestyle modifications. Future studies should focus on the impact of small support group meetings for overcoming the survivor’s medical and psychological issues.
